# Development of a Competitive Chemiluminescent Enzyme Immunoassay Based on Recombinant FMDV VP2 Protein for Rapid and Simultaneous Detection of Antibodies Against Foot-and-Mouth Disease Virus Serotypes O and A

**DOI:** 10.3390/vetsci13080774

**Published:** 2026-08-02

**Authors:** Shi’ao Bian, Zongzhe Yue, Long Zhou, Jun Ai, Diangang Han, Munib Ullah, Zhidong Zhang, Yanmin Li

**Affiliations:** 1 Key Laboratory of Animal Medicine of Sichuan Education Department, College of Animal and Veterinary Sciences, Southwest Minzu University, Chengdu 610041, China; 19856235652@163.com (S.B.); 15738535923@163.com (Z.Y.); zhoulongscu@163.com (L.Z.); liyanmin@swun.edu.cn (Y.L.); 2 Technology Center of Kunming Customs, Kunming 650228, China; 13769143048@163.com (J.A.); handiangang@outlook.com (D.H.); 3 Department of Clinical Studies, Faculty of Veterinary and Animal Sciences, PMAS-Arid Agriculture University, Rawalpindi 46300, Pakistan; drmunib15@gmail.com

**Keywords:** foot-and-mouth disease virus, recombinant VP2 protein, competitive chemiluminescent enzyme immunoassay, antibody detection

## Abstract

Timely detection of specific antibodies against foot-and-mouth disease virus (FMDV) is critical to effective prevention and control of the disease. In this study, we developed a competitive chemiluminescent enzyme immunoassay (cCLEIA) based on recombinant FMDV VP2 protein (FMDV-VP2 cCLEIA) for simultaneous detection of antibodies against FMDV serotypes O and A in serum samples. Using a cut-off of 35% percentage inhibition (PI), the FMDV-VP2 cCLEIA achieved a relative sensitivity of 101.5% and a relative specificity of 102% compared with commercial FMDV serotypes O and A liquid-phase blocking ELISA (LPBE) kits. The assay showed high specificity for detection of antibodies against FMDV serotypes A and O, with no cross-reactivity observed against positive sera for classical swine fever virus, porcine reproductive and respiratory syndrome virus, and Senecavirus A. Both intra-batch and inter-batch coefficients of variation were below 15%, confirming excellent assay reproducibility. The testing of 40 field serum samples yielded a positive detection rate of 95% using the FMDV-VP2 cCLEIA, compared with 92.5% using the FMDV serotypes O and A LPBE kits. In conclusion, the FMDV-VP2 cCLEIA developed is a rapid, sensitive assay for simultaneous detection of antibodies against FMDV serotypes O and A, which may offer a valuable tool for foot-and-mouth disease (FMD) surveillance and control programs.

## Simple Summary

Timely detection of foot-and-mouth disease virus (FMDV)-specific antibodies is critical to disease prevention and control. In this study, a competitive chemiluminescent enzyme immunoassay based on recombinant FMDV VP2 protein (FMDV-VP2 cCLEIA) was developed for simultaneous detection of antibodies against FMDV serotypes O and A. With a cut-off of 35% percentage inhibition (PI), the assay achieved a relative sensitivity of 101.5% and a relative specificity of 102%. It showed no cross-reactivity with sera known to be antibody-positive for multiple swine viruses, with both intra- and inter-batch coefficients of variation below 15%. For 40 field serum samples, its positive detection rate reached 95%, outperforming commercial liquid-phase blocking ELISA (LPBE) kits.

## 1. Introduction

Foot-and-mouth disease (FMD), caused by FMD virus (FMDV), remains one of the most economically devastating viral diseases affecting cloven-hoofed animals, including cattle, sheep, and pigs [1,2], which imposes severe restrictions on international trade of livestock and animal products, resulting in huge economic losses in endemic regions [3,4]. FMDV belongs to the genus Aphthovirus within the family Picornaviridae and exists as seven immunologically distinct serotypes (O, A, C, Asia 1, SAT 1, SAT 2, and SAT 3) [5,6]. There is no cross-protection between serotypes, alongside substantial intratypic antigenic diversity [5,7], which poses a major challenge for FMD vaccination strategies and diagnosis [8,9]. FMDV serotype O is the most prevalent, followed by FMDV serotype A, while FMDV serotype C has not been reported since 2004 [10]. FMDV SAT1-3 are predominantly found in sub-Saharan Africa, and Asia 1 is restricted to Asia. This complex epidemiological situation requires diagnostic assays capable of detecting antibodies against multiple circulating serotypes to enable effective surveillance and control of the disease [11,12].

Serological detection of antibodies against FMDV structural proteins is essential to effective FMD prevention and control [13,14]. Accurate and timely serological assays enable surveillance of infection, and confirmation of disease-free status for international trade [12,15] and post-vaccination serological monitoring [16,17]. Furthermore, serological surveys are indispensable to demonstrating freedom from infection in the context of international trade and for zoning purposes in FMD control programs [18,19]. However, current serological assays have critical limitations. The virus neutralization test (VNT), widely regarded as the gold standard for serotype-specific antibody detection, requires live virus and high-biocontainment facilities, making it logistically complex and inaccessible to many laboratories [20,21]. Liquid-phase blocking ELISAs (LPBEs) are prone to non-specific false positives, and both VNT and LPBE are labor-intensive, time-consuming, and requiring separate assays for each serotype of interest [21,22]. The need for serotype-specific reagents increases costs and complexity, particularly in regions where multiple serotypes co-circulate [11]. Lateral flow immunochromatographic assays (LFIAs) enable rapid pen-side detection but are qualitative, less sensitive, and unable to quantify antibody levels [23,24], limiting their utility for detailed immune response profiling. Furthermore, FMDV non-structural protein (NSP) ELISAs enable differentiation of infected from vaccinated animals (DIVA) but fail to detect vaccine-induced antibodies, limiting their utility for vaccine efficacy monitoring [25,26,27,28]. These drawbacks hinder rapid surveillance, timely outbreak response, and large-scale vaccination monitoring. This underscores the need for an assay which integrates multi-serotype reactivity, high sensitivity, specificity and rapid turnaround [11,12,29].

FMDV VP2 is a highly immunogenic capsid protein with conserved epitopes across all seven FMDV serotypes [30]. Unlike VP1, the most variable capsid protein, VP2 harbors three classes of conserved epitopes: linear epitopes at residues 67–78 (BC loop) and 115–126 (DE loop) [31]; a universal N-terminal epitope (residues 1–5) recognized by antibodies against all seven serotypes [9]; and conformational epitopes exposed by VP2’s structural flexibility [32]. These conserved regions enable VP2 to serve as a universal antigen for pan-serotype detection of FMDV antibodies [29]. Recombinant VP2 (rVP2) expressed in prokaryotic systems has demonstrated high immunoreactivity with sera from vaccinated and infected animals across multiple FMDV serotypes. A VP2-based indirect enzyme-linked immunosorbent assay (ELISA) achieved 100% sensitivity and 98.3% specificity against serotypes A, O, and SAT2 [33,34], while a VP2 peptide ELISA demonstrated 99% sensitivity and 93% specificity for broad serotype detection [33]. Nevertheless, these assays remain constrained by prolonged turnaround time and suboptimal specificity, particularly in field settings where rapid results are critical to outbreak containment [11,24].

Chemiluminescent enzyme immunoassay (CLEIA) combines the high sensitivity of chemiluminescent detection with robust specificity [35,36], offering superior performance compared with traditional ELISA. This technology provides enhanced sensitivity, a broader dynamic range, and reduced turnaround time due to its high signal-to-noise ratio [37]. In recent years, CLIA has been successfully developed for the detection of antibodies against various viral pathogens, including FMDV serotype-specific antibodies [38,39,40], highlighting its potential as a rapid, sensitive alternative to conventional assays.

In this study, we aimed to develop an FMDV-VP2 cCLEIA based on recombinant FMDV VP2 protein for simultaneous detection of antibodies against FMDV serotypes A and O and then evaluate its diagnostic performance in comparison with commercial LPBE kits for FMDV serotypes O and A.

## 2. Materials and Methods

### 2.1. Serum Samples

A panel of 173 serum samples archived with defined background was used for development and validation of the assay. Among these, 90 serum samples were FMDV antibody-negative, obtained from healthy animals with no history of infection and confirmed negative by commercial ELISA kit (Lanzhou Veterinary Research Institute Biotechnology Co., Ltd., Lanzhou, China). These negative samples comprise 32 bovine sera, 41 porcine sera and 17 ovine sera. The remaining 83 samples were FMDV antibody-positive, obtained from vaccinated animals, with antibody status verified by commercial ELISA kits, and included 38 porcine FMDV O/A bivalent antibody-positive sera, 16 FMDV O antibody-positive sera (8 bovine sera and 8 sheep sera) and 29 FMDV A antibody-positive sera (12 bovine sera, 6 porcine sera and 11 ovine sera). All serum samples were stored at −80 °C for long-term preservation prior to analysis.

### 2.2. Preparation of HRP-Labeled MAb Against FMDV

A mouse monoclonal antibody (MAb) specific to FMDV archived in our laboratory was purified from hybridoma culture supernatants by affinity protein G column chromatography (Bio-Rad, Hercules, CA, USA). The purified MAb was then conjugated with horseradish peroxidase (HRP), using an HRP conjugation kit according to the manufacturer’s instruction (Abcam, Waltham, MA, USA). The resulting HRP-labeled MAb (FMDV MAb-HRP) was aliquoted and stored at −20 °C until use.

### 2.3. Preparation of the Purified Recombinant VP2 Protein

The pET-28a plasmid encoding the FMDV VP2 gene (650 bp, O/BY/CHA/2010, GenBank: JN998085), preserved in our laboratory, was transformed into *E. coli* BL21 (DE3). Protein expression was induced with 0.8 mM isopropyl β-D-1- thiogalactopyranoside (IPTG) at 37 °C for 4 h. Cells were harvested by centrifugation (6000× *g*, 10 min, 4 °C), re-suspended in ice-cold PBS, and subjected to two rounds of ultrasonication with lysozyme treatment between rounds. The recombinant FMDV VP2 protein (rVP2) was purified from the supernatant using HisPur Ni-NTA Spin Columns (Thermo Scientific, Waltham, MA, USA) per the manufacturer’s protocol. SDS-PAGE was used to confirm a single ~27 kDa band, consistent with the predicted size, demonstrating that high-purity rVP2 was obtained via Ni-NTA affinity chromatography. The purified rVP2 was aliquoted and stored at −80 °C until use.

### 2.4. Development of FMDV-VP2 cCLEIA

The reaction conditions for the FMDV-VP2 cCLEIA were optimized using checkerboard titration, in which rVP2 coating concentration, dilution buffer, FMDV MAb-HRP dilution and incubation time were varied in parallel. The optimal conditions were selected based on the greatest difference in the percent inhibition (PI) values between FMDV-positive and -negative sera. Briefly, rVP2 was diluted to various concentrations with Tris-buffered saline (TBS; pH 7.3) and added to each well of a chemiluminescence microplate (Costar, Richmond, VA, USA) at 100 µL/well, followed by overnight incubation (≥16 h) at 4 °C. After washing three times with PBST (PBS containing 0.05% Tween-20), 100 µL/well of blocking buffer was added and incubated at 37 °C for 1 h, followed by three additional PBST washes. Subsequently, positive control, negative control, and test serum samples diluted in 3% BSA in PBST were added at 50 µL/well, followed immediately by 50 µL/well of FMDV MAb-HRP diluted in 3% BSA in PBST. The mixture was then incubated at 37 °C for 15–60 min. Antigen control wells contained FMDV MAb-HRP and 3% BSA in PBST diluent (without serum), and blank control wells contained only 3% BSA in PBST. After washing with PBST, 100 µL of luminol chemiluminescent substrate (PlasMarker Biotechnology Co., Ltd., Suzhou, China) was added to each well and incubated for 1 min at room temperature in the dark. The luminescence value at 435 nm was measured using an immunoassay analyzer (PerkinElmer, Springfield, IL, USA). PI was calculated using the following formula: PI % = [1 − (serum sample luminescence value − blank control luminescence value)/(antigen control luminescence value-blank control luminescence value)] × 100.

### 2.5. Determination of Cut-Off Value, Dsn and Dsp

A panel of sera (*n* = 173) with known background was used to establish assay performance parameters. Firstly, the cut-off value for the FMDV-VP2 cCLEIA was calculated as the mean PI of all negative samples (*n* = 90) plus two standard deviations (SDs). With this cut-off, diagnostic sensitivity (Dsn) and diagnostic specificity (Dsp) were determined. Dsn is the proportion of known positive samples correctly identified by the FMDV-VP2 cCLEIA, calculated as: Dsn (%) = (number of VP2-cCLEIA positive samples/total number of known positive samples) × 100. Dsp is the proportion of known negative samples correctly identified by the FMDV-VP2 cCLEIA, calculated as: Dsp (%) = (number of VP2-cCLEIA negative samples/total number of known negative samples) × 100. In addition, relative sensitivity and relative specificity were calculated by comparing the FMDV-VP2 cCLEIA results to those obtained with the commercial LPBE kit (Lanzhou Veterinary Research Institute Biotechnology Co., Ltd., Lanzhou, China), using the same 90 negative and 83 positive serum samples. Relative sensitivity and specificity were calculated as follows: relative sensitivity (%) = (FMDV-VP2 cCLEIA positive samples/LPBE positive samples) × 100; relative specificity (%) = (FMDV-VP2 cCLEIA negative samples/LPBE negative samples) × 100.

### 2.6. Analytical Sensitivity and Cross-Reaction

The analytical sensitivity of the FMDV-VP2 cCLEIA was determined and compared to that of the commercial LPBE kit using two-fold serial dilutions of an FMDV serotype O antibody-positive serum. The endpoint titer was defined as the highest serum dilution yielding a PI value ≥ the established cut-off. Analytical sensitivity was expressed as the reciprocal of this endpoint dilution.

To evaluate the assay cross-reactivity, the FMDV-VP2 cCLEIA was tested against a panel of positive sera including FMDV O and A, classical swine fever virus (CSFV), porcine reproductive and respiratory syndrome virus (PRRSV) and senecavirus A (SVA). Cross-reactivity was defined as a PI value ≥ the cut-off for any serum sample.

The intra- and inter-batch repeatability of the FMDV-VP2 cCLEIA was assessed using six serum samples (three positive and three negative). Repeatability was considered acceptable when both intra-batch and inter-batch coefficients of variation were below 15%.

### 2.7. Evaluation of Diagnostic Performance

The FMDV-VP2 cCLEIA and the commercial LPBE kit were used in parallel to test 40 field serum samples collected from pig farms which were archived in our laboratory. The diagnostic agreement between the two assays was evaluated by calculating Cohen’s kappa coefficient.

## 3. Results

### 3.1. Optimization of FMDV-VP2 cCLEIA

**Figure 1:**
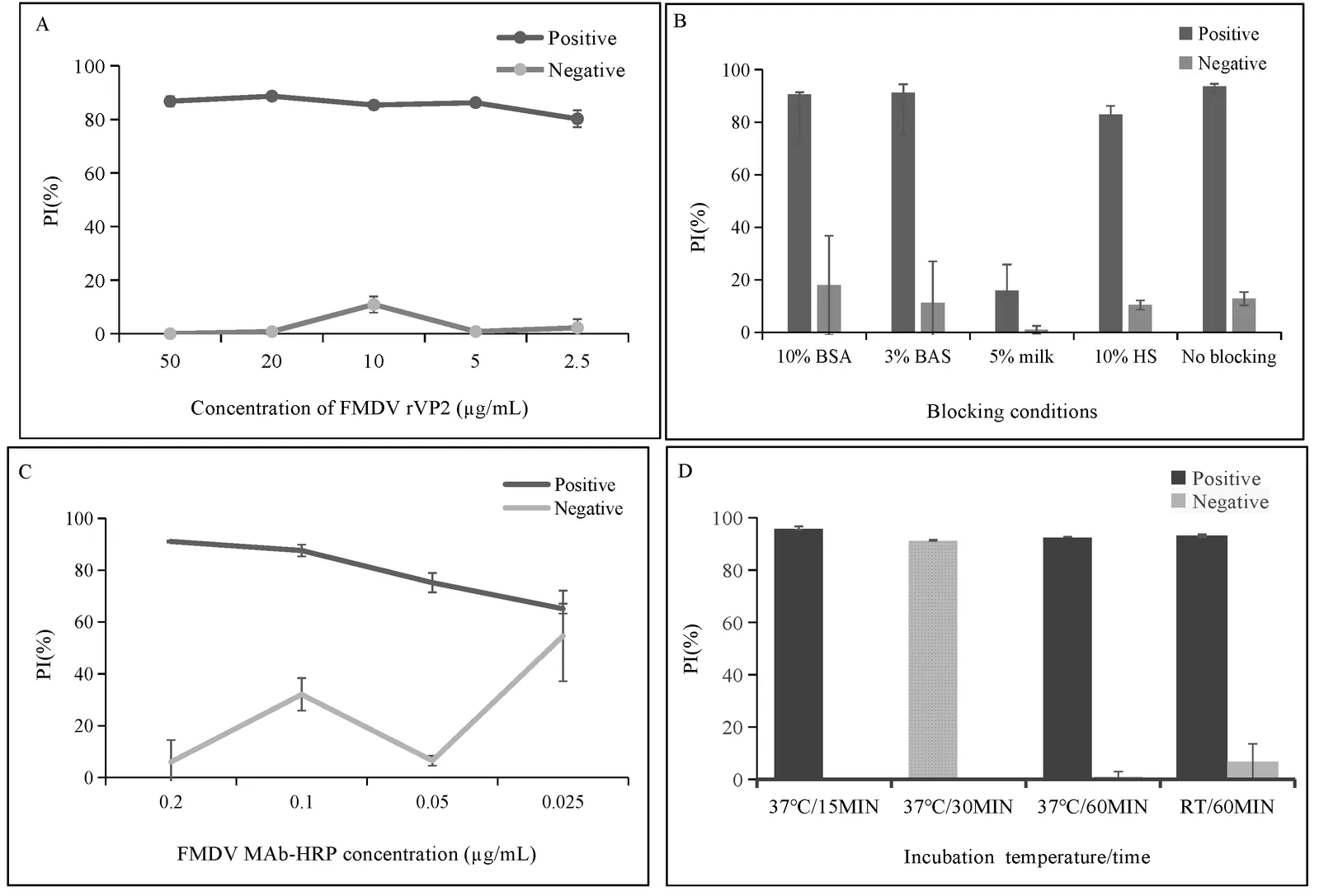
Optimization of FMDV-VP2 cCLEIA. (**A**) Screening of the optimal concentration of rVP2 protein. (**B**) Optimization of blocking conditions. (**C**) Determination of the optimal concentration of HRP-labeled FMDV monoclonal antibody (FMDV MAb-HRP). (**D**) Determination of incubation temperature and time. The data points represent mean values, and error bars represent standard deviation (SD). HRP = horseradish peroxidase.

**Figure 2:**
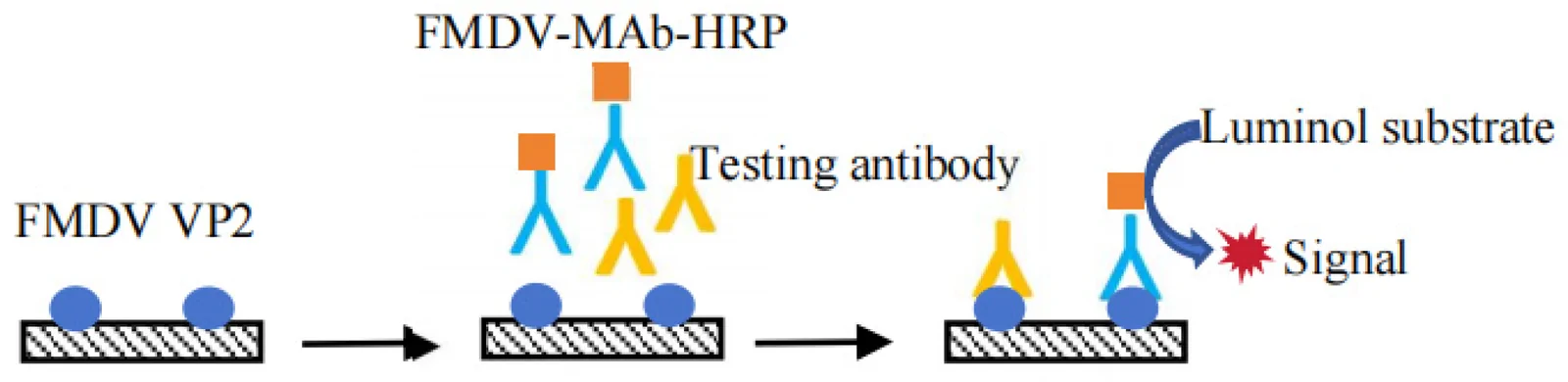
Schematic illustration of the FMDV-VP2 cCLEIA for detection of FMDV antibody. Arrows indicate the sequential steps of the reaction. Step 1: FMDV VP2 antigen is immobilized on the solid phase. Step 2: FMDV-MAb-HRP and testing antibody compete for binding to VP2. Step 3: Luminol substrate is added, and the HRP-catalyzed reaction produces a chemiluminescent signal. MAb = monoclonal antibody; HRP = horseradish peroxidase.

To maximize assay performance, key reaction parameters were systematically optimized to achieve the greatest difference in PI values between FMDV-positive and -negative sera (Figure 1). The optimized conditions were as follows: coating concentration of VP2 antigen, 5 µg/mL; FMDV MAb-HRP concentration, 0.2 µg/mL; incubation time, 15 min at 37 °C; no blocking step required; dilution buffer, 3% BSA in PBST. Under these conditions, the test turnaround time was 20 min. The developed FMDV-VP2 cCLEIA procedure is briefly shown in Figure 2.

### 3.2. Determination of Cut-Off Value, Dsn and Dsp

**Figure 3:**
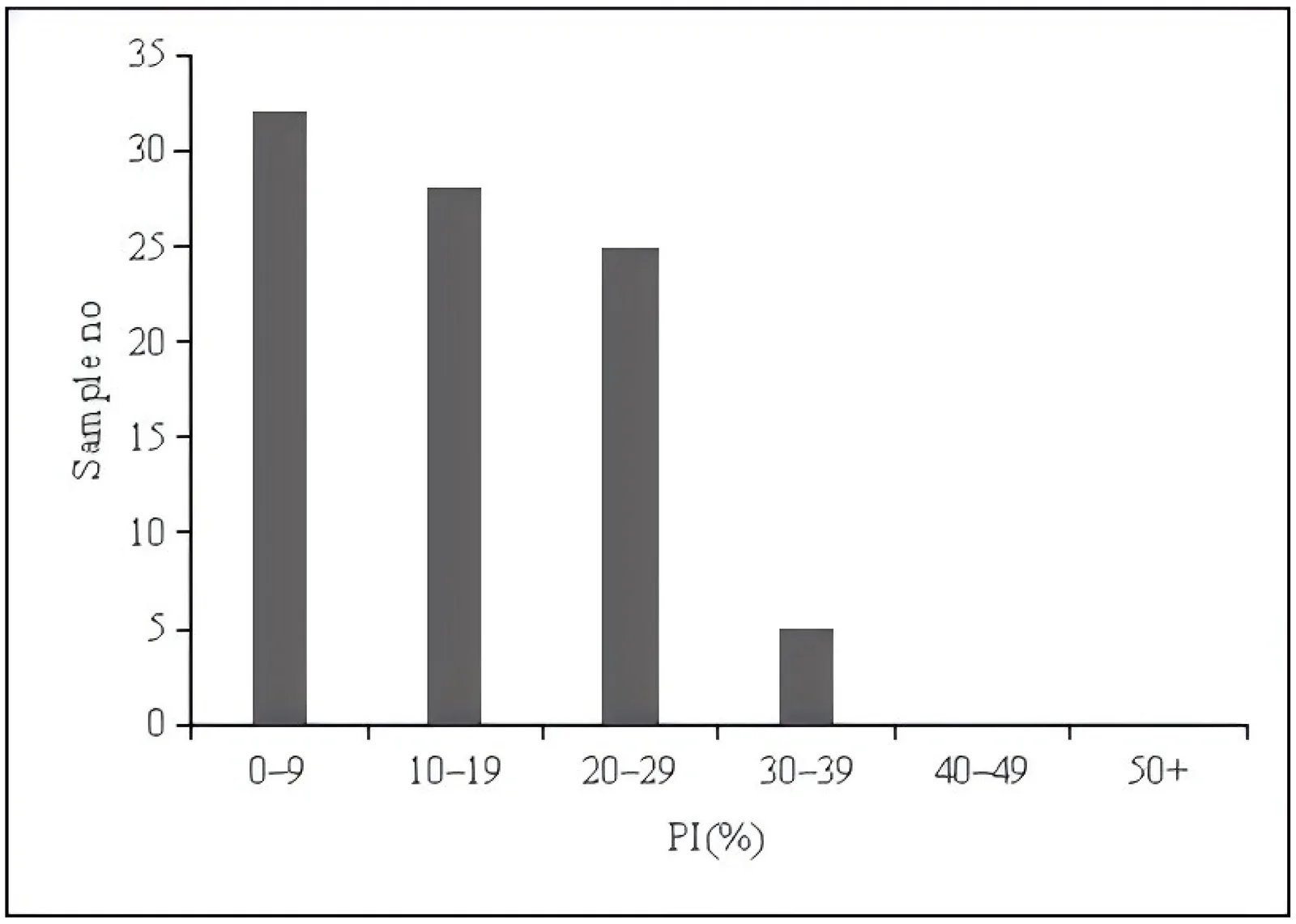
Distribution of PI % for FMDV antibody-negative sera.

To establish a cut-off for the FMDV-VP2 cCLEIA, the PI values of the 90 confirmed negative samples were measured. The mean PI and one SD for these negative samples tested were 14.93% and 10.08%, respectively (Figure 3). The cut-off value was defined as the mean PI of all negative samples + 2 SD. This yielded a cut-off PI of 35.%. The interpretation criteria were defined as follows: PI > 35: FMDV antibody-positive; PI ≤ 35%: FMDV antibody-negative. With this cut-off, FMDV-VP2 cCLEIA had a Dsn of 100% (83/83 known positive samples correctly identified) and a Dsp of 100% (90/90 known negative samples correctly identified).

### 3.3. Relative Sensitivity and Specificity Compared with LPBE Kits

To further validate the assay performance, the 173 serum samples were tested in parallel using FMDV O/A LPBE kits as the reference standard. In the FMDV O LPBE, serum samples with optical density at 450 nm (OD_450_) equal to or greater than the cut-off value of 0.5685 are interpreted as negative for the antibody; in the FMDV A LPBE, serum samples with OD_450_ equal to or greater than the cut-off value of 0.728 are interpreted as negative for the antibody. For FMDV O-positive sera (*n* = 54, including 38 O/A bivalent-positive sera and 16 O-positive sera), the relative sensitivity of the FMDV-VP2 cCLEIA was 100% (54/54 positive samples detected by both assays); for FMDV A-positive sera (*n* = 67, including 38 O/A bivalent-positive sera and 29 A-positive sera), the relative sensitivity of the FMDV-VP2 cCLEIA was 101.5% as 67/67 positive samples were identified as positive by the FMDV-VP2 cCLEIA, while 66/67 positive samples were identified as positive by the FMDV O/A LPBE kits. When considering all FMDV O- and A-positive samples (*n* = 83), the FMDV-VP2 cCLEIA correctly identified 83/83 positive samples, whereas the FMDV O/A LPBE kits detected 82/83 positive samples, resulting in a relative sensitivity of the FMDV-VP2 cCLEIA of 101% (Table 1A). For all FMDV O- and A-negative samples (*n* = 90), the VP2-cCLEIA correctly identified 90/90 negative samples, while the LPBE kits identified 88/90 negative samples as negative, yielding a relative specificity of the FMDV-VP2 cCLEIA of 102% (Table 1B).

### 3.4. Analytical Sensitivity, Cross-Reactivity and Repeatability

**Figure 4:**
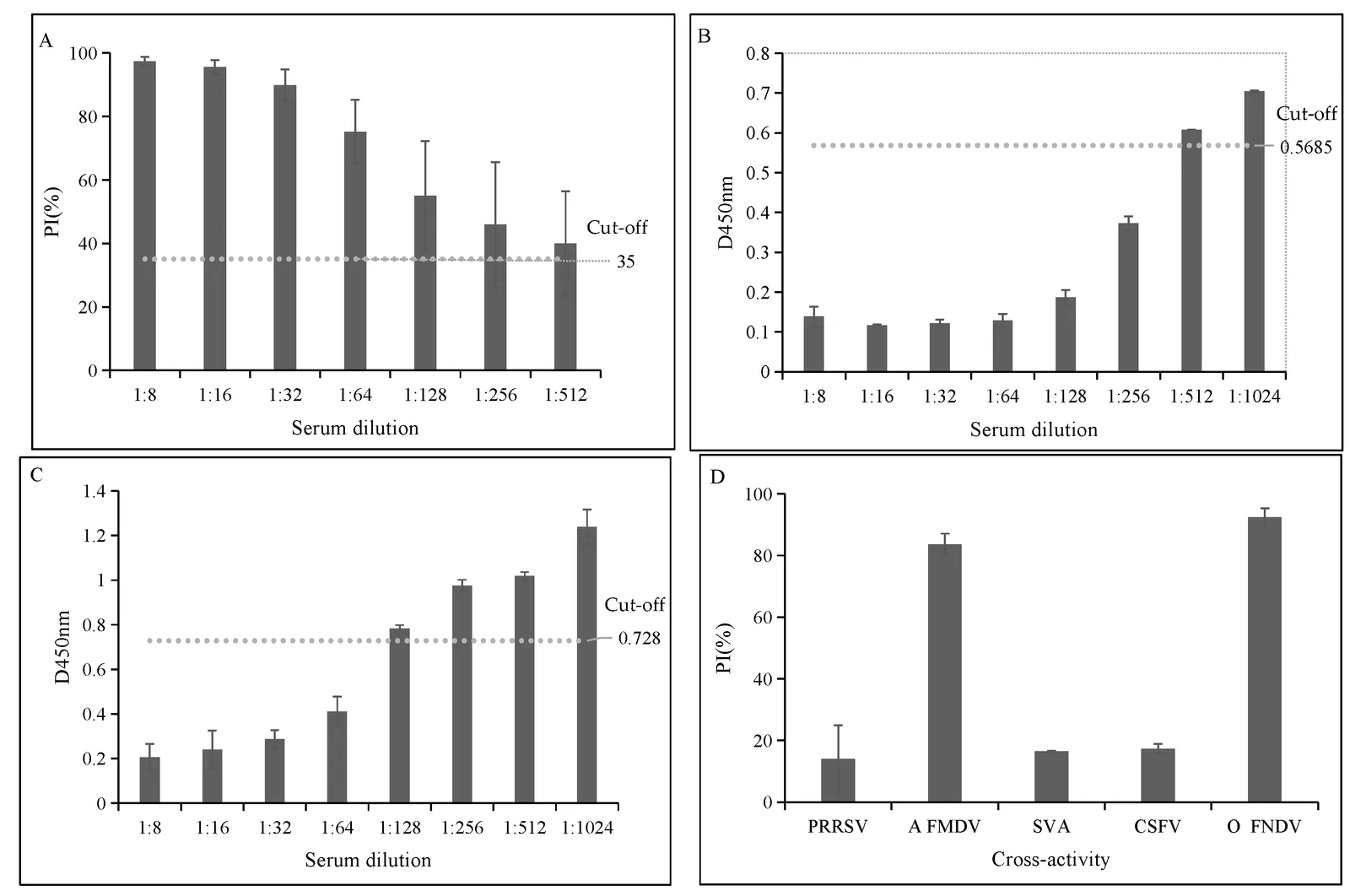
Analytical sensitivity and cross-reactivity of the FMDV-VP2 cCLEIA. (**A**) Analytical sensitivity of the cCLEIA. Positive when PI > 35. (**B**) Analytical sensitivity of the FMDV O LPBE. According to the manufacturer’s instructions, serum samples with optical density at 450 nm (OD_450_) equal to or greater than the cut-off value of 0.5685 are interpreted as negative for the antibody. (**C**) Analytical sensitivity of the FMDV A LPBE. According to the manufacturer’s instructions, serum samples with OD_450_ equal to or greater than the cut-off value of 0.728 are interpreted as negative for the antibody. (**D**) Cross-reactivity detection.

Analytical Sensitivity: To assess the limit of detection (LOD) of the FMDV-VP2 cCLEIA, an FMDV serotype O/A bivalent-positive serum was serially diluted two-fold (from 1:8 to 1:1024) and tested in parallel with commercial FMDV O and A LPBE kits. The endpoint titer based on discrete dilution points was defined as the highest serum dilution yielding a positive result (PI > 35% for FMDV-VP2 cCLEIA; OD_450_ < 0.5685 for FMDV O LPBE; OD_450_ < 0.728 for FMDV A LPBE). The result showed that the FMDV-VP2 cCLEIA detected antibody activity at a dilution of 1:512 (Figure 4A), whereas the commercial LPBE kit detected titers of 1:256 for serotype O (Figure 4B) and 1:64 for serotype A (Figure 4C).

Cross-Reactivity: To evaluate the cross-reaction, the FMDV-VP2 cCLEIA was tested against positive sera from FMDV O, FMDV A, CSFV, PRRSV and SVA, with three replicates per serum type. FMDV O- and A-positive sera tested positive (PI > 35%), while CSFV-, PRRSV-, and SVA-positive sera yielded PI values below the cut-off (<30%), indicating no cross-reactivity (Figure 4D). These results confirm that the FMDV-VP2 cCLEIA is highly specific for FMDV serotypes O and A antibodies, with no cross-reactivity to other common porcine viral pathogens.

Repeatability: To assess assay repeatability, three FMDV-positive samples and one negative serum sample were tested by the FMDV-VP2 cCLEIA. The results demonstrated that the intra-batch coefficient of variation ranged from 0.06% to 3.87%, while the inter-batch coefficient of variation ranged from 0.53% to 10.87%. Both intra-batch and inter-batch coefficients of variation were below 15%, confirming excellent reproducibility of the assay (Table 2).

### 3.5. Evaluation with Field Serum Samples

To validate the diagnostic performance of the FMDV-VP2 cCLEIA, 40 field-collected serum samples were tested in parallel with commercial FMDV O and A LPBE kits. As shown in Table 3, the FMDV-VP2 cCLEIA identified 38 positive samples and 2 negative samples, resulting in a positive detection rate of 95%. In comparison, the commercial FMDV O LPBE kit detected 31 positive samples and 9 negative samples, yielding a positive detection rate of 77.5%, and the FMDV A LPBE kit detected 32 positive samples and 8 negative samples, yielding a positive detection rate of 80%. Overall, the combined FMDV O/A LPBE kits identified 37 positive and 3 negative samples, corresponding to a positive detection rate of 92.5%. To quantify the level of agreement between the assays, Cohen’s kappa coefficient was calculated to be 0.787, indicating substantial agreement and confirming the reliability of the FMDV-VP2 cCLEIA for field sample testing.

## 4. Discussion

FMD remains a transboundary threat to global livestock industries, with significant economic and food security implications [3,4]. Serological detection of FMDV antibody is essential to effective FMD control and prevention [13,14]. However, traditional methods such as VNT and ELISA are constrained by either reliance on live viruses or long turnaround times, as well as inherent serotype specificity [20,21]. These limitations hinder rapid, large-scale surveillance, especially in regions where multiple FMDV serotypes co-circulate. To address these gaps, we developed an FMDV-VP2 cCLEIA for simultaneous detection of FMDV antibody against serotypes O and A, which may offer a valuable tool for FMD surveillance and control programs.

The selection of VP2 as the coating antigen was driven by its unique structural and immunological properties [33]. VP2 harbors conserved linear and conformational epitopes which are recognized by antibodies across FMDV serotypes. This cross-reactivity forms the basis for detection of antibodies across multiple FMDV serotypes [30,31,32]. Previous studies have validated the utility of VP2 for pan-serotype FMDV antibody detection [32,33,34]. A VP2-based indirect ELISA achieved 100% sensitivity and 98.3% specificity against serotypes A, O, and SAT2 [33], while a VP2 peptide ELISA demonstrated 99% sensitivity and 93% specificity for multi-serotype detection [32]. This serotype-independent capability is particularly valuable for endemic regions with co-circulating FMDV serotypes, such as sub-Saharan Africa and Southeast Asia [10,12]. By replacing serotype-specific assays with a pan-serotype test, surveillance workflows are streamlined, reducing costs and turnaround times, which has critical advantages during outbreak responses [11,24]. Our findings align with these reports, showing that rVP2 as the coating antigen in the cCLEIA exhibited high specificity for FMDV serotypes A and O, with no cross-reactivity against sera positive for CSFV, PRRSV and SVA.

Compared with the LPBE, the FMDV-VP2 cCLEIA developed offers comparable diagnostic performance due to the high signal-to-noise ratio of chemiluminescent detection. The assay detected antibodies in a bivalent O/A-positive serum with 1.4-fold and 5.7-fold increases in sensitivity compared with commercial FMDV O and A LPBEs, respectively. The relative sensitivity and relative specificity of the FMDV-VP2 cCLEIA were 101% and 102%, respectively, when compared with commercial FMDV O and A LPBEs. These values exceeding 100% may be a consequence of LPBE not being a perfect gold standard and the higher analytical sensitivity and specificity of the chemiluminescent platform, which can detect antibody levels below the LPBE detection threshold. This high sensitivity mirrors observations in magnetic particle-based CLIA (MP-CLIA) for FMDV serotype A antibodies [40] and is particularly impactful for early post-vaccination monitoring, where low antibody titers are critical indicators of protective immunity. Validation with 40 field serum samples confirmed the real-world utility of the FMDV-VP2 cCLEIA, yielding a 95% positive detection rate, which is 2.5% higher than the commercial LPBE. Further evaluations incorporating VNT as a complementary reference standard would help resolve these discrepancies and provide a more definitive assessment of assay accuracy. Additionally, in the FMDV-VP2 cCLEIA, there is no blocking step required, and the competitive reaction step and signal readout require only 20 min, which is much quicker than the commercial LPBE, enabling rapid decision making during outbreaks. Despite the promising performance of the FMDV-VP2 cCLEIA, several limitations should be acknowledged in this study. First, although the assay demonstrated high sensitivity and specificity for serotypes O and A, its ability to detect antibodies against other epidemiologically important FMDV serotypes (including C, Asia 1, SAT 1, SAT 2, and SAT 3) was not experimentally validated due to the limited availability of positive sera for these serotypes in our laboratory. Given that VP2 contains conserved epitopes across all FMDV seven serotypes, it is reasonable to expect that the assay would also detect antibodies against these FMDV serotypes. However, future studies are required to evaluate its performance in sera against Asia 1, SAT1-3 and C. Second, the field validation was conducted using a relatively small porcine sample set (*n* = 40), and the serum panel was geographically restricted with limited information on sample origin. Therefore, larger-scale, multi-regional studies incorporating well-characterized sera from diverse geographical sources and multiple host species are required to further confirm the robustness and generalizability of the assay. In addition, the cross-reactivity evaluation in this study is limited, and future studies should include a broader range of vesicular and non-vesicular pathogens that may cause similar clinical signs.

## 5. Conclusions

The FMDV-VP2 cCLEIA developed in this study enables rapid, sensitive, and simultaneous detection of antibodies against FMDV serotypes O and A. Following further validation with larger and more geographically diverse sample sets and after experimental evaluation against other FMDV serotypes (C, Asia 1, and SAT 1–3), this assay could serve as a complementary tool for global FMD prevention and control in regions where serotypes O and A co-circulate.

## Figures and Tables

**Table 1:** Relative sensitivity (**A**) and relative specificity (**B**) of the FMDV-VP2 cCLEIA compared with the LPBE kits.

(**A**)				
**Assays**		**FMDV O/A LPBE** **Kits**		
		**Positive**	**Negative**	**Total**
FMDV-VP2 cCLEIA	Positive	82	1	83
	Negative	0	0	1
	Total	82	1	83
(**B**)				
**Assays**		**FMDV O/A LPBE** **Kits**		
		**Positive**	**Negative**	**Total**
FMDV-VP2 cCLEIA	Positive	0	0	0
	Negative	2	88	90
	Total	2	88	90

Note: (**A**) Relative sensitivity of the FMDV-VP2 cCLEIA kit compared with the LPBE kits. (**B**) Relative specificity of the FMDV-VP2 cCLEIA compared with the LPBE kits.

**Table 2:** Repeatability of the FMDV-VP2 cCLEIA.

Intra-batch			
Sample No.	Average PI (%)	SD	CV (%)
1	96.3	0.02	0.27
2	97.4	0.1	0.17
3	97.7	0.06	0.06
4	7.3	0.2	3.87
Inter-batch			
Sample No.	Average PI (%)	SD	CV (%)
1	93.3	0.1	1.65
2	96.3	0.5	0.54
3	96.4	0.5	0.53
4	7.9	0.8	10.87

Note: Samples 1–3 represent FMDV-positive sera (PI approximately 96–97%); Sample 4 represents FMDV-negative serum (PI approximately 7–8%). CV = coefficient of variation; SD = standard deviation.

**Table 3:** Comparison of the FMDV-VP2 cCLEIA with the LPBEs.

Assays		FMDV O/A LPBE Kits		
		Positive	Negative	Total
FMDV-VP2 cCLEIA	Positive	37	1	38
	Negative	0	2	2
	Total	37	3	40
	Kappa Value	0.787		

## Data Availability

The original contributions presented in this study are included in the article. Further inquiries can be directed to the corresponding author.

## Abbreviations

The following abbreviations are used in this manuscript:

FMDV	foot-and-mouth disease virus
CLIA	chemiluminescent immunoassay
PI	percent inhibition
LPBE	liquid-phase blocking ELISA kits
Dsn	diagnostic sensitivity
Dsp	diagnostic specificity
VNT	virus neutralization test
CLEIA	chemiluminescent enzyme immunoassay
CSFV	classical swine fever virus
PRRSV	porcine reproductive and respiratory syndrome virus
SVA	senecavirus A
IPTG	isopropyl thiogalactoside
MAb	monoclonal antibody
NSP	non-structural protein
DIVA	differentiating infected from vaccinated animals
LFIA	immunochromatographic assays
